# Point-of-Care Veterinary Diagnostics Using Vis–NIR Spectroscopy: Current Opportunities and Future Directions

**DOI:** 10.3390/ani16030401

**Published:** 2026-01-28

**Authors:** Sofia Rosa, Ana C. Silvestre-Ferreira, Rui Martins, Felisbina Luísa Queiroga

**Affiliations:** 1 Animal and Veterinary Science Research Centre (CECAV), University of Trás-os-Montes and Alto Douro (UTAD), 5001-801 Vila Real, Portugal; sofiaasrosa99@gmail.com (S.R.); aferreir@utad.pt (A.C.S.-F.); 2 Department of Veterinary Science, University of Trás-os-Montes and Alto Douro (UTAD), 5001-801 Vila Real, Portugal; 3 Associated Laboratory for Animal and Veterinary Science (AL4AnimalS), 1300-477 Lisboa, Portugal; 4 Institute for Systems and Computer Engineering, Technology and Science (INESC TEC), 4200-465 Porto, Portugal; 5 Center for the Study of Animal Sciences (CECA-ICETA), University of Porto, 4099-002 Porto, Portugal

**Keywords:** near-infrared spectroscopy, point-of-care diagnostics, veterinary medicine, One Health, artificial intelligence

## Abstract

Visible-Near-Infrared (Vis-NIR) spectroscopy, spanning approximately 400 to 2500 nm, is an innovative technology with growing relevance for diagnostics performed at the point of care (POC). This review explores the potential of Vis-NIR in veterinary medicine, highlighting its advantages over complex techniques like Raman and Fourier transform infrared spectroscopy (FTIR) by being rapid, non-invasive, reagent-free, and compatible with miniaturized, portable devices. The methodology involves directing a broadband light source, often using LEDs, toward the sample (e.g., blood, urine, faeces), collecting spectral information related to molecular vibrations, which is then analyzed using chemometric methods. Successful veterinary applications include hemogram analysis in dogs, cats, and Atlantic salmon, and quantifying blood in ovine faeces for parasite detection. Key limitations include spectral interference from strong absorbers like water and hemoglobin, and the limited penetration depth of light. However, combining Vis-NIR with Self-Learning Artificial Intelligence (SLAI) is shown to isolate and mitigate these multi-scale interferences. Vis-NIR spectroscopy serves as an important complement to centralized laboratory testing, holding significant potential to accelerate clinical decisions, minimize stress on animals during assessment, and improve diagnostic capabilities for both human and animal health, aligning with the One Health concept.

## Simple Summary

This study explores how Visible-Near-Infrared (Vis-NIR) spectroscopy can transform veterinary diagnostics at the point of care (POC). Unlike more complex laboratory techniques, Vis-NIR is fast, non-invasive, and compatible with portable, low-cost POC devices. By measuring how light interacts with biological samples, this technology provides immediate biochemical results without the need for chemical reagents. Although challenges remain, combining Vis-NIR with Self-Learning Artificial Intelligence (SLAI) is essential to improve accuracy and filter out technical interferences. This synergy not only speeds up diagnosis but also makes healthcare more accessible, directly supporting the One Health initiative.

## 1. Introduction

The advancement of medical technology has driven a continuous demand for diagnostic methods that combine high accuracy with rapid turnaround times. In clinical practice, especially in emergency scenarios, the speed of diagnostic data acquisition is a critical determinant of therapeutic success. Historically dependent on centralized laboratories, diagnostics are undergoing a paradigm shift towards point-of-care (POC) settings, allowing for immediate assessment in clinics, field environments, or even at home. This decentralization is not merely a technical innovation but a strategic move to integrate diagnostic precision directly into the primary care workflow [1].

In the veterinary context, the clinical necessity for rapid diagnostics is further underscored by the inability of animal patients to verbally communicate their symptoms. Consequently, clinicians must rely on objective physiological data to support practical, real-time decision-making in the field. Beyond operational efficiency, current trends suggest a narrowing gap between human and animal healthcare technologies. This convergence facilitates a multidisciplinary environment where shared methodologies enhance diagnostic capabilities across species, fundamentally aligning with the One Health framework [2].

### 1.1. Purpose and Perspective

This review aims to explore the potential and growing relevance of visible-short-wave-near-infrared (Vis-NIR) spectroscopy as a diagnostic tool in veterinary medicine, particularly within point-of-care (POC) settings. While Vis-NIR spectroscopy has been widely studied in human health [3,4,5,6,7,8] and agriculture [9,10,11,12,13,14], its use in veterinary clinical practice, especially in fast and practical settings, is still expanding. This work seeks to support that growth and help reinforce its value in animal diagnostics.

Our goal is to offer veterinarians, researchers, and professionals from both medical and technological fields an accessible overview of how this technology can be adapted to animal health care. This includes both in vivo and ex vivo applications, with a focus on low-cost and portable setups that can be used directly in clinics or field environments. In doing so, we aim to highlight how spectroscopy, traditionally limited to research or highly specialized labs, is becoming a viable option for veterinary practice.

This review also introduces a new perspective: Vis-NIR spectroscopy is not just a technical tool, but part of a bigger movement toward healthcare that is more connected, faster, and interdisciplinary. Learning from human medicine, we believe that point-of-care Vis-NIR devices can help spot problems earlier, monitor animal health more closely, and guide better decision-making.

To better understand the unique advantages of Vis-NIR, it is helpful to consider how it compares with other spectroscopic techniques already used in veterinary medicine. Raman spectroscopy, for instance, has already been used in veterinary contexts such as urine analysis, disease identification, and detection of physiological compounds [15,16,17]. It offers high molecular specificity and is useful for identifying biomarkers in biological samples [18,19]. However, its use in vivo is limited by background fluorescence and by the complexity of the equipment, which typically requires controlled laboratory conditions [20].

Another technique is Fourier transform infrared spectroscopy (FTIR), which is widely used for detailed chemical analysis of ex vivo samples, such as tissues and biological fluids [21]. It has already been applied in veterinary medicine to quantify immunoglobulins in serum, support the diagnosis of infectious diseases, and detect changes in joint fluids associated with inflammatory processes, among others [22,23,24,25]. Despite its potential, the direct application of FTIR to live animals is still hampered by the strong interference of water in the spectra and the size and complexity of the equipment involved, which restricts its use to highly controlled laboratory environments [26].

Similarly, fluorescence spectroscopy has found broad use in veterinary diagnostics and research, enabling rapid measurement of hormone levels, diagnosis of inflammatory conditions, and assessment of reproductive cell functionality [27,28,29]. Despite its versatility and sensitivity, most clinical and veterinary applications of fluorescence spectroscopy rely on fluorescent labels or probes, require invasive sample collection, and depend on relatively bulky or delicate detection systems, which constrain its routine use in veterinary practice [30].

Faced with these limitations, Vis-NIR spectroscopy stands out by combining several practical advantages that make it particularly well-suited for veterinary medicine. It is non-invasive, rapid, and compatible with portable, miniaturized devices, which is ideal for both clinical and field environments. Covering a broad spectral range, it enables real-time monitoring of multiple physiological and biochemical parameters without requiring reagents or complex laboratory preparation [31,32,33]. These features position Vis-NIR spectroscopy as a promising technology, especially in POC veterinary contexts, where simplicity, speed, and portability are essential.

### 1.2. History and Concept

Vis-NIR spectroscopy, which spans wavelengths from approximately 400 to 2500 nm [34], is a natural evolution of near-infrared spectroscopy (NIR) (750–2500 nm) [35]. The roots of this technique go back to the early 1800s when William Herschel first discovered infrared radiation. Yet, despite this early finding, it took more than a century for its analytical value to truly emerge. That moment came in 1960 when Karl Norris, working at the US Department of Agriculture (USDA), used NIR spectroscopy to measure moisture in grains and seeds. His approach was fast, practical and non-destructive, and quickly drew the attention of the industry. Together with Phil Williams, Norris helped prove that NIR could be a reliable tool for agricultural monitoring, especially after its successful use in the rapid analysis of wheat shipments in Canada. By the late 1970s, John Shenk had expanded its application to forage analysis, paving the way for a national network dedicated to predicting the nutritional quality of animal feed. Still, it was not until 1983 that Professor David Wetzel famously described NIR as a “sleeping technique,” suggesting that much of its potential remained untapped. Since then, NIR has become a widely trusted method, valued not just for its speed but for its ability to support routine monitoring of key parameters across agriculture and beyond [36].

The integration of the visible spectrum (400–750 nm) [35] into the NIR range led to the development of Vis-NIR spectroscopy, expanding its analytical capabilities.

Since the 1980s, advances in fiber optics, smaller light sources like light-emitting diodes (LEDs), and compact detectors have made it possible to create portable Vis-NIR devices. These instruments have taken spectroscopy out of the lab and into the field, allowing for quick, non-invasive, and reagent-free analysis across many industries [1].

While much of the push for miniaturizing this technology has come from human healthcare, veterinary medicine is now starting to catch up. Recent research shows that portable Vis-NIR devices can accurately estimate blood parameters in cats and dogs without the need for reagents or complicated sample preparation [1,33]. These promising developments suggest that Vis-NIR spectroscopy could become a practical, easy-to-use diagnostic tool right at the point of care in veterinary clinics.

### 1.3. How Is Vis-NIR Spectroscopy Performed?

A typical Vis-NIR spectral detection system or instrument always includes a light source, a monochromator, a detector, and some optical accessories [37].

The light source plays a crucial role in the Vis-NIR spectroscopy detection system, providing the energy needed to illuminate the samples. Traditionally, tungsten-halogen lamps have been used due to their wide spectral coverage. However, they have important limitations, such as excessive heat emission and a short lifespan. For this reason, LEDs have emerged as a promising alternative, featuring higher electro-optical conversion efficiency, much longer service life, insensitivity to vibration and no risk of causing radiation burns. Despite their narrow bandwidth (30–50 nm), LEDs have been successfully integrated into portable instruments for biomedical and clinical applications. More recently, light sources based on multiple LEDs have been developed to generate adjustable spectra, facilitating the calibration and adaptability of detection systems. However, challenges remain, such as the complexity of the electronic controls and the uniform distribution of light, making it difficult to integrate them into Vis-NIR detection systems [38].

**Figure 1:**
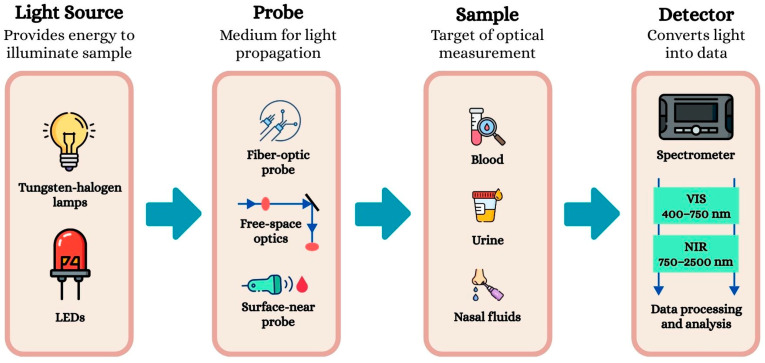
Overview of the Vis-NIR spectroscopy process and system architecture. The diagram illustrates the physical sequence of signal acquisition: starting with the energy emission from the light source (LEDs or Tungsten-halogen lamps), followed by light propagation through the probe (fiber optics or free space); the interaction with the sample (blood, urine, or fluids) via transmittance or reflectance; and finally, the detection by the spectrometer.

In practice, Vis-NIR spectroscopy (Figure 1) involves directing a broadband light source towards the target area. The emitted light can be delivered through fiber-optic cables, free-space optics, or directly from a probe positioned close to the surface of interest [35]. The interaction between light and sample can occur via reflectance, transmittance, or interacting modes, depending on the optical setup and the nature of the sample [38]. The portion of light that is collected after this interaction carries spectral information related to the molecular vibrations and physical properties of the medium. The distance between the light source and the detector, as well as the intensity and pressure of illumination, can influence the quality and accuracy of the collected spectra. The detected light is then analyzed to extract information about the characteristics of the illuminated area [35].

**Figure 2:**
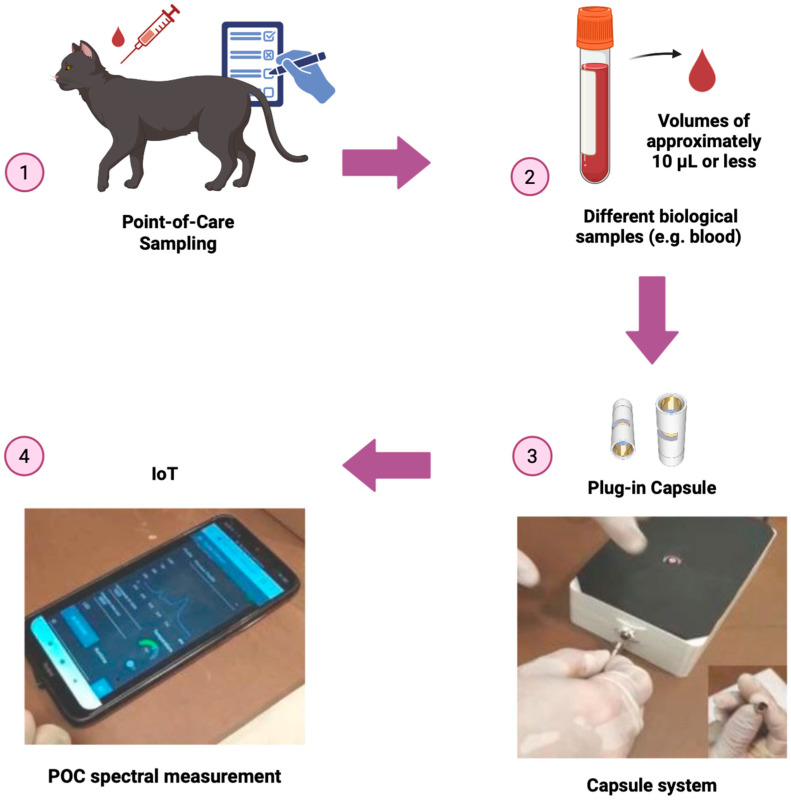
Integrated POC sampling and analytical workflow cycle. The diagram details the four main stages of the diagnostic process: (1) Point-of-care sampling in the feline patient; (2) handling of minimal sample volumes (~10 μL); (3) insertion of the specialized Plug-in Capsule into the Capsule System; and (4) real-time IoT-based spectral measurement and result visualization.

In practice, these technical principles are implemented in POC IoT platforms using miniaturized spectrometer modules, such as the Hamamatsu C12666MA (socket adapter, Hamamatsu, Japan) or USB-based systems like the Ocean Insight STS-vis. These devices manage multiple light sources, including power LEDs (4500 K) with optimized temperature and modulation. The optical setup utilizes transmittance fiber optics, where the biological sample is placed in a plug-in reusable capsule. These capsules, designed with opposing mirrors and a path length of 5 mm, allow for high-precision analysis using minimal sample volumes of approximately 10 μL or less, enabling real-time spectral registration (Figure 2). While these physical components are responsible for signal acquisition, the analytical processing is handled by Self-Learning Artificial Intelligence (SLAI). This distinction is crucial, as the hardware captures the raw multi-scale interference, while the SLAI unscrambles these spectral patterns to provide quantitative blood parameters [32].

### 1.4. The Relevance of Vis-NIR Spectroscopy and the Rise of POC Diagnostics

As technology and medicine evolve, the demand for accurate and rapid diagnostic methods is steadily increasing. In emergency care and medical atmospheres, time is a critical factor in treatment success. In this context, innovative technologies such as Vis-NIR spectroscopy are revolutionizing the approach to medical diagnostics.

By enabling rapid, reagent-free, and non-invasive analysis of biological samples, Vis-NIR spectroscopy offers a powerful alternative to traditional laboratory methods by capturing physical (e.g., scattering, reflectance, shadows) and molecular interactions (e.g., absorbance, fluorescence) signals from different components (e.g., blood) at distinctive wavelengths in the visible and near-infrared regions [31,32,33]. These technologies have become particularly relevant in recent years, as healthcare systems worldwide seek more sustainable, rapid, and decentralized diagnostic solutions, especially in low-resource or remote settings.

This technological potential becomes even more significant when merged with the concept of POC settings. POC diagnostics refers to procedures performed close to the patient, without the need to send samples to centralized laboratories. This approach reduces waiting times for results, facilitates clinical decision making and improves the patient experience, which is very valued in both human and veterinary medicine [39,40].

In this context, the convergence of Vis-NIR spectroscopy and POC testing represents a step forward in diagnostic innovation, combining speed and accessibility, as Vis-NIR-based POC systems have demonstrated the ability to provide real-time clinical parameters from micro-volumes of samples, with minimal preparation and high efficiency [1,31,41].

Such a combination reflects a broader shift in healthcare: one that highlights fast diagnostics and patient-centered approaches. By bridging speed with precision, and accessibility with analytical depth, Vis-NIR spectroscopy is poised to become a cornerstone in the next generation of diagnostic tools.

### 1.5. The Lights and Shadows of Vis-NIR

Although Vis-NIR spectroscopy is a promising tool for POC diagnostics, it is important to recognize its limitations as a first step toward improvement.

One of the primary limitations of Vis-NIR spectroscopy concerns the way light interacts with biological samples, particularly regarding scattering and penetration. Scattering effects, including geometric, Mie, and Rayleigh scattering, leave unique scattering patterns in the spectra for different cell types with their different sizes and shapes, and operate simultaneously at distinctive scales, affecting each wavelength differently. This scattering has traditionally been observed as a systematic effect that has to be corrected and is often ignored as being unrelated to chemical composition. Thus, scatter correction algorithms are usually applied as pre-processing steps. Also, chemometric and artificial intelligence methods have largely focused on absorbance or transmittance modelling, often neglecting the scattering information contained in the spectral data [1]. Another physical constraint of Vis-NIR spectroscopy is the limited penetration depth of light in biological tissues. Even though NIR light penetrates deeper than visible light, it typically reaches only up to 5–7 mm, which may be insufficient to access deeper structures or pathophysiological processes in certain diagnostic scenarios [42].

Another important limitation comes from the physical characteristics of the samples themselves. Differences in how samples are prepared, for example, using synthetic mixtures versus doped production samples, can create distinct scattering patterns that affect the spectra. These effects are often linked to variations in particle size or how uniform the sample is, and they are not always fully corrected by common preprocessing methods like derivatives or normalization. Because of this, it can be difficult to validate or apply calibration models reliably across different types of samples [43].

Water and temperature are two important factors that can strongly influence Vis-NIR spectroscopy in biological samples. Water is a strong absorber, especially in the NIR region, which can mask or overlap with signals from other components, complicating spectral interpretation. Meanwhile, temperature variations affect both the absorption characteristics and the overall spectral response, introducing variability that, if not properly controlled, can result in false positives or negatives. Together, these factors highlight the need for careful sample handling and environmental control to ensure reliable and accurate measurements [43,44].

Molecular specificity also poses a significant challenge for Vis-NIR spectroscopy. Due to overlapping spectral bands, it can be difficult to distinguish between compounds with similar chemical structures. This lack of specificity sometimes requires the use of hybrid techniques or dedicated sensors to accurately identify and quantify individual components within complex biological samples [45].

In blood samples, which are among the most commonly used biological samples for diagnostic purposes, one of the major challenges is the difficulty in extracting accurate information from blood spectra due to multi-scale interference, in which spectral bands overlap, and matrix effects such as pH variations and scattering distort the signal. This interference spreads both qualitative and quantitative information across all wavelengths. A key aspect of this lies in the fact that dominant information in blood spectra comes from highly absorbent constituents in the Vis-NIR region, such as hemoglobin (Hgb) and bilirubin. Since red blood cells (RBC) are the most abundant cells in the blood, and Hgb strongly absorbs light, their signal tends to dominate the spectra. Other elements present in smaller amounts or with weaker signals can overlap with these dominant features, making it harder to clearly detect and interpret everything. Additionally, the global covariance in biological datasets is unstable and multidimensional due to sample variability, often leading to deviations from the Beer–Lambert law (BLL), complicating correct quantification of blood constituents [1,32].

Common approaches to mitigate spectral interference focus on reducing sample complexity through technologies such as sample separation, lab-on-a-chip devices, or the use of biochips with biological specificity (e.g., immunological reactions). However, these methods do not take full advantage of the information provided by spectroscopy, which provides data on a wide range of compounds in a single measurement. To address this, SLAI has been developed to turn multi-scale interference into an advantage by using covariance mode (CovM) search to isolate different types of interferences, providing quantitative information while reducing dimensionality, thereby improving the interpretation of interferences and the quantification of constituents with greater precision, especially for pure constituents, where interference is absent [1,32,33].

**Figure 3:**
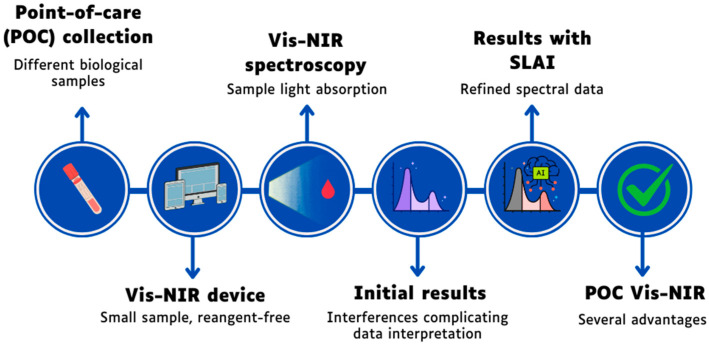
Analytical workflow and logic of the POC Vis-NIR diagnostic system. This figure illustrates the transition from the physical collection to the results. It highlights the role of the Vis-NIR device in reagent-free sampling and the core challenge of initial results, where complex interferences complicate data interpretation. The subsequent phase shows the results with SLAI, where Self-Learning Artificial Intelligence (SLAI) unscrambles multi-scale spectral interferences to provide refined, accurate diagnostic outcomes, showcasing the final advantages of the POC Vis-NIR technology.

Vis-NIR spectroscopy combined with SLAI has great potential for improving POC diagnostics (Figure 3). However, fully understanding and integrating all aspects of the spectral signal, including scattering and matrix effects, will be key to making these technologies more reliable and widely used in clinical settings.

Miniaturization of Vis-NIR devices improves portability and convenience for POC diagnostics but often reduces sensitivity and spectral resolution. This reduction can limit the ability to detect subtle signals in biological samples. Furthermore, the high costs associated with manufacturing and maintaining these devices, along with the need for specialized training, limit their accessibility, especially in resource-limited settings. Therefore, it is essential to find a balance between innovation, cost, and performance to make this technology viable for widespread clinical use [37,46].

## 2. Vis-NIR Spectroscopy: A Diagnostic Tool for All

Despite its limitations, Vis-NIR spectroscopy is a versatile tool with the potential to democratize access to health technologies. However, this democratization depends on a critical transition from high-cost, specialized laboratory equipment to affordable and robust devices capable of performing under “field conditions”. While moving towards a more generalized approach, the technology must overcome significant barriers, such as ensuring sensor stability against environmental variables (e.g., temperature and humidity) and simplifying calibration models so they can be reliably operated by non-specialists outside of controlled environments. By delivering real-time, reagent-free results, it proves especially valuable in contexts where timely decisions are essential. In both human and veterinary medicine, it serves as a practical alternative to conventional laboratory testing, offering a quicker way to assess samples such as blood and respond with greater precision.

This shared potential aligns with the One Health perspective, moving beyond the mere shared use of technology toward an emergent comparative approach. By considering data and diagnostic principles from one domain to inform new methodologies in the other, Vis-NIR spectroscopy fosters a deeper integration of human and animal health monitoring. This cross-species synergy allows for the translation of spectral biomarkers, where insights gained from diverse physiological models contribute to a more holistic and innovative diagnostic framework.

Even so, laboratory diagnostics, particularly in clinical pathology, remain the gold standard for analyzing biological samples like blood, urine, or faeces. These laboratories are essential for producing accurate, validated results, supported by rigorous protocols and quality control. Their role is fundamental, especially in complex or uncertain cases that require in-depth analysis. However, relying exclusively on centralized laboratories can delay diagnosis and treatment, particularly in remote or under-resourced areas. The time required for sample transport, batch processing, and access to specialized equipment and staff often slows clinical decisions. In this regard, POC technologies like Vis-NIR can help close the gap. While they do not replace laboratory testing, they extend their reach by enabling immediate, on-site analysis. This not only removes logistical barriers but also accelerates the path from sample to action, supporting faster, more accessible care.

In human medicine, Vis-NIR spectroscopy has been applied to a wide range of biological samples, demonstrating the versatility of this technology. Blood has been one of the most extensively studied matrices with applications including hemoglobin quantification [4], glucose monitoring [5], evaluation of hemodialysis materials (assessing how different materials interact with blood components during dialysis) [47] and prediction of serum bilirubin levels [48]. The ability to assess bilirubin, a key indicator in liver function and other health conditions, exemplifies the broader role of biomarkers in modern diagnostics, which are essential for detecting, monitoring and managing diseases, making their accurate measurement a priority. Also, more recently, Vis-NIR has been used to evaluate the severity of post-COVID conditions [49], highlighting its adaptability to emerging health challenges where new diagnostic indicators continue to be discovered.

Beyond blood, nasal fluids and mucus have shown great promise in detecting influenza-related infections [7,8], offering important insights into virus-specific inflammatory responses. These studies are especially significant given the global impact of viral diseases and the urgent need for fast, reliable diagnostic tools.

Despite focusing primarily on biological fluids, it is important to highlight that Vis-NIR spectroscopy has proven effective across various biological matrices, reinforcing its value as a flexible and informative diagnostic approach. It has also been applied to histological samples to quantify prognostic markers in lymphomas [50], offering new possibilities for cancer evaluation and monitoring. In orthopedics, it has helped differentiate healthy from osteoarthritic cartilage [6], supporting earlier diagnosis and better treatment planning. Other studies have explored applications in living tissue, teeth, and dental prostheses to assess skin type, monitor microcirculation, and measure natural tooth color [3].

In veterinary medicine, although the number of studies remains more limited, the diversity of applications already reported reflects the wide-ranging potential of Vis-NIR spectroscopy. Blood has also been the most explored biological fluid, with successful applications in dogs, cats and salmon for evaluating complete blood counts, including leukocyte and platelet quantification, as well as hemogram analysis [1,31,32,33,41,51].

Fecal samples have also been investigated, particularly for the screening of pathological markers associated with parasitology. While Vis-NIR spectroscopy has shown potential in this area, it should be noted that such applications often act as screening tools rather than definitive diagnostic methods. In clinical practice, these results must be integrated into a differential diagnosis, as the technology may detect the consequences of a pathological process (e.g., hemorrhage) without necessarily identifying the specific underlying cause among multiple etiologies. One study demonstrated the capacity of Vis-NIR to quantify blood in ovine faeces, enabling early detection of *Haemonchus contortus* infections [52], a significant parasitic threat in small ruminants, and given the impact of parasitic diseases on animal health and productivity, the development of fast, non-invasive diagnostic tools represents an important step forward. In the dairy sector, differences in the composition of ovine milk associated with different feeding systems have also been characterized [53], showing how it can support dietary assessments while also proving useful across multiple aspects of animal management.

In line with its applications in human medicine, Vis-NIR spectroscopy has also been explored for use in solid tissues within veterinary contexts. It has been used to distinguish gestational sacs from surrounding abdominal structures in rabbits [54] and to assess tissue composition during cartilage repair in porcine osteochondral samples [55]. Altogether, these studies reinforce the adaptability of Vis-NIR to different species, sample types, and clinical contexts in veterinary settings.

In Table 1, we can see the various applications of Vis-NIR spectroscopy in both human and veterinary medicine, particularly in the analysis of biological fluids, further illustrating its potential for monitoring and diagnosing specific conditions across different species.

Although several parallels can be drawn with human medicine, the application of Vis-NIR spectroscopy in veterinary contexts also brings distinct challenges and considerations. The wide variety of species in veterinary practice leads to significant differences in blood cell shape and composition, which can directly impact the spectral data. For example, dog RBC are larger with a central pale area, while cat RBC are smaller and flatter. Additionally, the relationship between RBC, Hgb, and HTC is species-specific, as dog blood contains more Hgb per RBC and exhibits stronger absorption in the hemoglobin bands between 430–630 nm compared to cat blood. These physiological differences must be carefully considered when designing diagnostic models and interpreting the spectral data. Each species presents its unique characteristics, meaning that diagnostic approaches that work for one may not be directly applicable to another. However, this challenge is also an opportunity to refine and adapt Vis-NIR spectroscopy to meet the diverse needs of veterinary medicine, ultimately improving its accuracy and utility in animal diagnostics. Many diagnostic tools used in human medicine are either unavailable or cost-prohibitive in animal health. Vis-NIR spectroscopy, especially when miniaturized and integrated with cloud-based AI platforms, could represent an affordable alternative that improves the standard of care in general practice. This is particularly valuable in species where diagnostic development has lagged or where sampling is difficult, such as in small animals, exotics, or wildlife [32].

Furthermore, veterinary diagnostics face the issue of environmental variation, with factors such as diet, climate and even the time of day influencing the results of tests. For instance, the composition of blood and other biological samples can vary significantly depending on an animal’s diet or stress levels, making it essential to develop calibration models that take these variables into account. While this adds complexity to the application of Vis-NIR spectroscopy, it also pushes the boundaries of the technology, driving further innovation and refinement.

Nevertheless, Vis-NIR remains a valuable tool in veterinary diagnostics, not just because of its analytical capabilities, but also because it reduces the need for repeated invasive tests, helping to minimize stress and discomfort for the animal during clinical assessments. This non-invasive approach is especially critical in veterinary settings, where reducing the physical and emotional toll on animals can significantly improve the quality of care. It also offers a potential for more frequent monitoring, allowing for early detection of health issues and more timely interventions, which can make a huge difference in the treatment outcomes. In this context, the clinical utility of Vis-NIR can be categorized into two distinct roles: pre-diagnostic screening and post-diagnostic monitoring. As a screening tool, its rapid nature allows for the assessment of large populations or asymptomatic individuals, identifying subclinical deviations that warrant further investigation. Conversely, as a monitoring tool, it provides a cost-effective way to track disease progression or therapeutic efficacy, enabling clinicians to make data-driven adjustments to treatment plans based on the real-time evolution of specific biomarkers.

The connection between these two fields through Vis-NIR opens up exciting possibilities for sharing knowledge. What we learn from veterinary diagnostics can be applied to human healthcare, and the reverse is also true, creating a collaborative space where both fields can develop together. As the technology continues to evolve, Vis-NIR spectroscopy has the potential to become an essential tool in both human and veterinary medicine, bridging the gap between them and improving diagnostic capabilities for both.

## 3. Conclusions

Vis-NIR spectroscopy has shown great promise as a diagnostic tool that is both versatile and accessible in human and veterinary medicine. Its key strengths lie in its ability to provide rapid, reagent-free, and non-invasive results. This makes it particularly valuable in situations where quick decision-making is essential, such as in remote locations or places where resources and access to traditional laboratory facilities are limited.

While clinical pathology laboratories remain the gold standard for detailed and complex analysis of biological samples, Vis-NIR spectroscopy serves as an important complement rather than a replacement. It helps to bring diagnostics closer to the point of care, enabling health professionals to act faster and with more confidence. This speed can be life-changing, allowing earlier detection of diseases, timely treatment, and ultimately better health outcomes for both humans and animals.

One of the exciting aspects of Vis-NIR is its adaptability across different species and sample types. This flexibility fits perfectly with the One Health concept, which highlights the deep connection between human, animal, and environmental health. Using Vis-NIR in both medical fields encourages collaboration and knowledge sharing, where insights gained from one can inform and improve practices in the other.

The diversity of animal species, with their unique physiological differences, presents challenges when applying Vis-NIR technology. Blood composition, cell size, and other factors vary greatly from species to species, requiring tailored calibration and interpretation. Environmental factors like diet, climate, and stress also influence test results, adding another layer of complexity. These challenges, however, are not obstacles but opportunities that push the technology forward, encouraging continuous innovation and refinement.

Moreover, Vis-NIR’s non-invasive nature means it can reduce the need for repeated blood draws or other stressful procedures in animals. This is particularly important in veterinary medicine, where minimizing discomfort and stress improves the overall quality of care and animal welfare. It also allows for more frequent monitoring, helping detect health changes early and improving the chances of successful treatment.

Looking to the future, ongoing advancements in Vis-NIR technology, especially when combined with cloud-based artificial intelligence and machine learning platforms, promise to make this tool even more accessible, affordable, and precise. However, it is essential to recognize the technical challenges of moving from specialized, single-parameter laboratory setups to generalized, multi-analyte POC devices. The simultaneous quantification of multiple parameters requires overcoming significant spectral interference and overlapping absorption bands. While advanced deconvolution algorithms are being developed to address these issues, further research is needed to ensure the robustness of these multi-parametric models in diverse clinical environments. This evolution could help bridge gaps in healthcare, particularly in underserved areas or with species where diagnostic tools are still lacking.

In conclusion, Vis-NIR spectroscopy holds real potential to transform diagnostics in both human and veterinary medicine. It offers a bridge between the two fields, opening up new possibilities for faster, more affordable, and less invasive health assessments. By continuing to develop and apply this technology with care and collaboration, we can improve outcomes and support healthier lives for people and animals alike.

## Figures and Tables

**Table 1:** Applications of Vis-NIR spectroscopy in biological fluid analysis in human and veterinary medicine.

Year	Sample Type	Application/Findings	Reference
**Human**			
1994	Blood	Identification of hemoglobin NIR absorption bands and demonstration of total hemoglobin quantification with high accuracy	[4]
2012	Nasal fluids	Rapid detection of Influenza A and B; high sensitivity and specificity	[7,8]
2015	Blood	Reagent-free glucose quantification; high accuracy compared to standard glucometers	[5]
2018	Blood	Real-time hemodialysis monitoring; compensation of spectral interference from tubing materials	[47]
2020	Blood (serum)	Prediction of bilirubin levels; rapid diagnosis of jaundice and liver dysfunction	[48]
2024	Blood	Classification and quantification of post-COVID condition (PCC) severity	[49]
**Animal**			
2018	Milk (Sheep)	Discrimination of milk properties and traceability of feeding systems	[53]
2020	Faeces (Sheep)	Quantification of occult blood in feces; screening for gastrointestinal hemorrhage as an indicator of *Haemonchus contortus* infection	[52]
2021	Blood (Dog and Cat)	Hemogram analysis, simultaneous prediction of hematological parameters in canine and feline whole blood samples	[32]
2021	Blood (Dog)	Precise quantification of total leukocytes and platelets	[31,41]
2022	Blood (Dog)	Total leukocyte count; high correlation with gold-standard flow cytometry	[33]
2023	Blood (Atlantic salmon)	Health monitoring in aquaculture; cellular counting of blood components	[51]
2024	Blood (Dog)	Refinement of cellular counting protocols; cellular counting of blood components	[1]

## Data Availability

No new data were created or analyzed in this study.
